# digiBONE: an automated tool for segmental Greulich-Pyle bone age assessment of Indian children and adolescents

**DOI:** 10.3389/fendo.2026.1757571

**Published:** 2026-03-11

**Authors:** Shreya Chakladar, Chirantap Oza, Shruti Mondkar, Tim R. J. Aeppli, Lars Sävendahl, Anuradha Khadilkar, Vaman Khadilkar, Pranay Goel

**Affiliations:** 1 Department of Biology, Indian Institute of Science Education and Research (IISER) Pune, Pune, India; 2 Hirabai Cowasji Jehangir Medical Research Institute (HCJMRI), Pune, India; 3 Division of Pediatric Endocrinology, Department of Women’s and Children’s Health, Karolinska Institutet, Stockholm, Sweden; 4 Pediatric Endocrinology Unit, Karolinska University Hospital, Stockholm, Sweden; 5 Department of Health Sciences, Savitribai Phule Pune University, Pune, India; 6 Jehangir Hospital, Pune, India

**Keywords:** Greulich-Pyle, bone age assessment, carpals, convolutional neural networks, full-hand radiographs, segmental skeletal maturity, short bones, wrist bones

## Abstract

**Introduction:**

Accurate bone age assessment (BAA) is essential for diagnosing and managing pediatric endocrine and growth disorders, as it reflects biological maturity beyond chronological age. The widely used Greulich–Pyle (GP) method estimates bone age by visually comparing full-hand radiographs with standardised reference images. Although widely used, this technique—and most automated systems based on it—assumes uniform skeletal maturation across the hand. In practice, however, skeletal maturation progresses at different rates in the anatomical segments of the hand under varied hormonal influences. This segmental variability may contribute to inter-observer inconsistency and diagnostic uncertainty. We developed digiBONE, a deep learning framework that models segment-specific skeletal maturation.

**Methods:**

Hand radiographs were segmented into anatomically coherent regions—short bones, carpals, and wrist—representing synchronously maturing bones. Separate convolutional neural networks (CNNs) were trained for each segment and for the full hand. Segmental predictions were combined with the full-hand model to generate a composite bone age prediction.

**Results:**

Integration of segmental maturity improved performance over full-hand-only models, achieving mean absolute differences (MAD) of 4.75 months for boys and 4.93 months for girls. In addition to improved accuracy, it revealed asynchrony between hand regions providing complementary maturity information beyond global estimates.

**Discussion:**

The full-hand model captures how a child’s overall maturity aligns with population norms, while, the segmental models explain how individuals of the same GP age differ biologically, improving interpretability and personalisation. digiBONE demonstrates that integrating biologically relevant segmental information into deep learning pipelines offers a scalable, automated bone age assessment solution applicable to Indian populations.

## 1 Background

Bone age is a clinical indicator used to assess a child’s growth status. The bones of the hand and wrist have a series of discrete growth phases depicting different maturity stages of the bone (1). The correlation between bone maturity and chronological age helps clinicians detect growth disorders, pubertal development, and guide them in making decisions about interventions (2–4). One of the oldest techniques used for assessing a child’s bone age is the Greulich and Pyle (GP) method. The GP atlas, developed in 1959, contains reference hand radiographs of Caucasian children under 19 years of age and their corresponding bone ages (1). A clinician compares a patient’s full hand X-ray with “gold standard” radiographs, as defined in the GP atlas, to determine the patient’s bone age. While this approach has long been considered the clinical benchmark, it is inherently limited by its dependence on subjective human interpretation, which brings about substantial rater variability. Inter- and intra-rater variability is a prominent concern of this manual method, which may sometimes lead to inconsistencies in diagnosis and treatment decisions (5, 6). In addition, manual evaluation is a labor-intensive and time-consuming process, which poses a considerable challenge in a clinical environment with a high volume of patients. Another prominenet BAA method, the Tanner-Whitehouse 3 (TW3) (7), grades the maturity of 13 predefined regions of interest (ROIs). Each ROI is assigned a maturity score, and the summed scores are converted into a skeletal age using standardized reference tables. The TW3 approach is often regarded as more reproducible and anatomically explicit than GP, but it is also considerably more complex and time-consuming, requiring substantial expertise and effort from trained radiologists (8). These practical limitations have constrained its widespread adoption in routine clinical workflows, despite its higher interpretability at the bone level. The limitations of both GP and TW3 have played a pivotal role in motivating the development of automated bone age assessment systems.

The automation of bone age assessment (BAA) has been dramatically accelerated by the recent release of the extensive, publicly accessible Radiological Society of North America (RSNA) pediatric bone age dataset and by advances in deep learning (9). In particular, convolutional neural networks (CNNs) have transformed the BAA landscape, shifting from knowledge- or rule-based (10–13) to data-driven learning (14, 15). BoneXpert (3) stands out as one of the earliest and most widely utilized automated commercial systems, utilizing classical machine learning to produce GP and Tanner-Whitehouse 3 (TW3) bone age estimates in less than 15 seconds per radiograph (14). When evaluated on the RSNA dataset, the software achieves root mean squared errors (RMSEs) of 8.16 and 6.24 months for GP-based predictions in males and females, respectively, and 6.12 and 5.88 months for TW3-based assessments. Furthermore, its validation on pediatric populations in India demonstrated good performance, with RMSEs of 6.36 and 4.68 months (GP method) and 5.64 and 4.80 months (TW3 method) for males and females, respectively (16). More recent automation strategies exemplify the transition to fully CNN-based systems. VUNO Med-BoneAge (https://www.vuno.co/en/boneage) is a CNN-based, commercially available system that has outperformed BoneXpert, especially in pre-pubertal female cohorts (17). A mean absolute difference (MAD), i.e. the average absolute error between the model’s prediction and the ground truth, of 6.8 months has been observed by Physis (https://www.16bit.ai/), another CNN-driven platform (14, 18). Ren et al. (19) used coarse and fine-attention maps of the hand X-ray images to train a regression CNN network and reported an MAD of 5.2 months on the RSNA dataset. These CNN-based approaches, which are made possible by the increasing availability of large-scale annotated datasets, achieve high accuracy and robustness while reducing reliance on handcrafted features and rule-based heuristics.

Early studies in automated bone age assessment predominantly adopted GP-based paradigms, using full-hand radiographs as input to deep learning models for direct age regression (20–23). These approaches demonstrated the feasibility of data-driven automation but treated the hand as a single anatomical unit. In parallel, several studies have automated the Tanner–Whitehouse (TW3) method by explicitly modeling bone-level maturity scores using deep learning, thereby introducing an inherently segmental perspective at the level of individual regions of interest. Wu et al. (8) proposed SVTNet, a fully automated TW3-based bone age assessment framework that localizes key skeletal regions using a CNN-based detector and classifies individual bones with a Vision Transformer, achieving MAD of 5.64 months. Son et al. (24) automated the TW3 protocol by detecting and classifying 13 skeletal regions using deep neural networks, reporting an MAD of 5.52 months. Zhang et al. (25) introduced SMANet, a multi-region ensemble framework for automated bone age assessment. The method explicitly partitions the hand into multiple anatomical regions (13 TW3-RUS regions and carpals), trains separate CNNs for each region, and combines their predictions through an ensemble strategy to achieve an MAD of 5.1 months. More recently, research has begun to explore segmental modeling beyond individual bones by partitioning the hand into broader anatomical regions (26, 27). For instance, Jung et al. (28) extracts important parts of the hand, such as the phalanges, metacarpals, carpals and wrist bones, from ‘unnecessary’ background; they obtain segment-wise predictions and average them to attain a MAD of 5.69 months. Iglovikov et al. (26) trained CNN models for carpals, metacarpals and proximal phalanges in addition to whole-hand model using the RSNA dataset and reported that the whole hand model achieved the lowest MAD of 6.1 months among all the models. Simu and Lal (29) segmented the hand bone region from its tissue regions to eliminate irrelevant information from the background. Li et al. (30) used a cascading bone region extraction network to sequentially extract relevant bone structures (using an attention-based strategy) for age prediction; MAD of 5.45 months on the RSNA dataset. While these methods highlight the technical benefits of segmental modeling, they primarily view segmentation as a computational step for better localization, with limited focus on the biological or developmental relevance of the segments. We note that the models were trained using *full-hand* labels; in particular, they did not use *segment-specific* labels.

However, it has long been recognized since the foundational work of Greulich and Pyle, that skeletal maturation does not occur uniformly across the various anatomical regions of the hand. Oza et al. (31) have suggested that hormonal influences play a role in the development of skeletal parts; for instance, sex hormones predominantly affect the maturation of tubular bones such as the radius, ulna, metacarpals, and phalanges, whereas the carpal bones follow a different developmental trajectory. Based on these differential growth patterns, they grouped the bones into three main anatomical “segments”: short bones (metacarpals and phalanges), carpals, and the wrist region (radius and ulna). These groupings are thus based on coherent maturation rates (within segments). Anatomical and hormonal variability may help explain the inter- and intra-rater variability observed in manual bone age assessment, particularly in cases where asynchronous maturity across segments poses significant challenges in estimating a single bone age. Building on this rationale, Chapke (32) trained a Densenet161 model on the RSNA dataset to predict segmental ages independently of the full-hand age. Chapke et al. (33) have argued that this so-called “segmental-GP BAA” has several attractive features, especially for interpretation. In this manuscript we continue with this strategy to present a neural network trained to predict segmental ratings for an Indian cohort.

We describe digiBONE, a novel automated bone age assessment framework to report segmental variability in skeletal maturity across the hand, and specifically adapted for pediatric populations in India. This design provides insight into CNN-driven decision-making by highlighting segment-wise differences in bone development. In particular, this especially helps in interpreting cases that do not readily conform to GP-defined archetypes. Further, we describe a weighting scheme to combine the segmental and full-hand predictions. This method will enable principled decision-making in cases of differential maturity.

## 2 Methods

### 2.1 Datasets

This study utilizes two datasets as follows:

RSNA dataset - We utilized the open-access Hand X-ray dataset from the RSNA 2017 bone age assessment challenge (9). It contains 14,036 X-ray images of Caucasian subjects, along with their corresponding bone age labels. **Table 1** summarizes the key statistics of the RSNA datasets, including sex-specific sample size, the average bone ages, and the overall age range covered. The reference RSNA ratings were determined by calculating the mean of the ratings from six independent clinicians using the GP method of bone age estimation. We treat this age as “ground truth labels” against which the models were trained.
HCJBA dataset - We additionally had access to 2,430 Hand X-rays of Indian Children obtained from Hirabai Cowasji Jehangir Medical Research Institute (HCJMRI), Pune, which were developed using microSCAN-HF diagnostics X-ray systems from Skanray Technologies. We refer to it as the HCJBA dataset. The key statistics of the HCJBA datasets, detailing the sex-specific sample sizes, mean bone ages, and age ranges, are summarized in **Table 1**. This dataset was subsequently partitioned into training and validation subsets for model fine-tuning and performance evaluation. Specifically, the male subset was split into 1,071 training and 179 validation samples, and the female subset into 1,011 training and 169 validation samples.

**Table 1:** Summary statistics of the bone age datasets showing sex-specific sample size, mean age and age-range.

Dataset	Sex	Dataset size	Mean ± SD (months)	Age range (months)
RSNA	Male	7606	135.3 ± 42.1	1–288
RSNA	Female	6430	118.0 ± 37.6	4–216
HCJBA	Male	1250	80.2 ± 29.5	36–216
HCJBA	Female	1180	101.4 ± 34.6	36–216

### 2.2 Segmental labeling of the hand

Each image was assigned full-hand and “segmental” GP labels. Segmental GP labels were obtained by isolating the region of interest (ROI) and masking all other hand regions to blind the clinician. The clinician then matched the isolated ROI to the corresponding region in the GP atlas. The bone age of the atlas image exhibiting the closest anatomical match was assigned as the segmental bone age for that ROI. This procedure of segmental labeling is a novel methodological extension to the standard GP method and is not part of a standard bone age rating method. All labeling was independently performed by two pediatric endocrinologists (VK and CO—25 years and 5 years of experience, respectively) to ensure consistency and reliability (31). Inter-rater reliability was high (quadratic-weighted Cohen’s *κ* = 0.81), with 65% exact agreement and 90% agreement within ±1 GP class.

### 2.3 Automated segmentation of hand X-Ray image

Chapke (32) trained a U-Net on the RSNA hand X-ray dataset by partitioning the full-hand image into its shortbone, carpal and wrist segments. However, when applied to HCJBA images, the segmentation models failed to recognize the ROIs, likely due to some underlying shift in the newer dataset. This “distribution shift” could be due to a mismatch in the underlying image statistics, protocols or population (34). The running mean and variance of the Batch Normalization layer learned by training on RSNA images could not generalize on the target dataset. To mitigate this, we employed test-time adaptation strategy in the segmentation models. Specifically, the running mean and variance parameters of the Batch Normalization layers learned during RSNA training were disabled at inference time. This allows normalization to be driven by the statistics of each input image rather than the source-domain distribution, thereby improving alignment with the target domain without requiring any additional learning or parameter updates with no additional computational latency. This approach successfully generated segment masks corresponding to the three ROIs.

**Figure 1:**
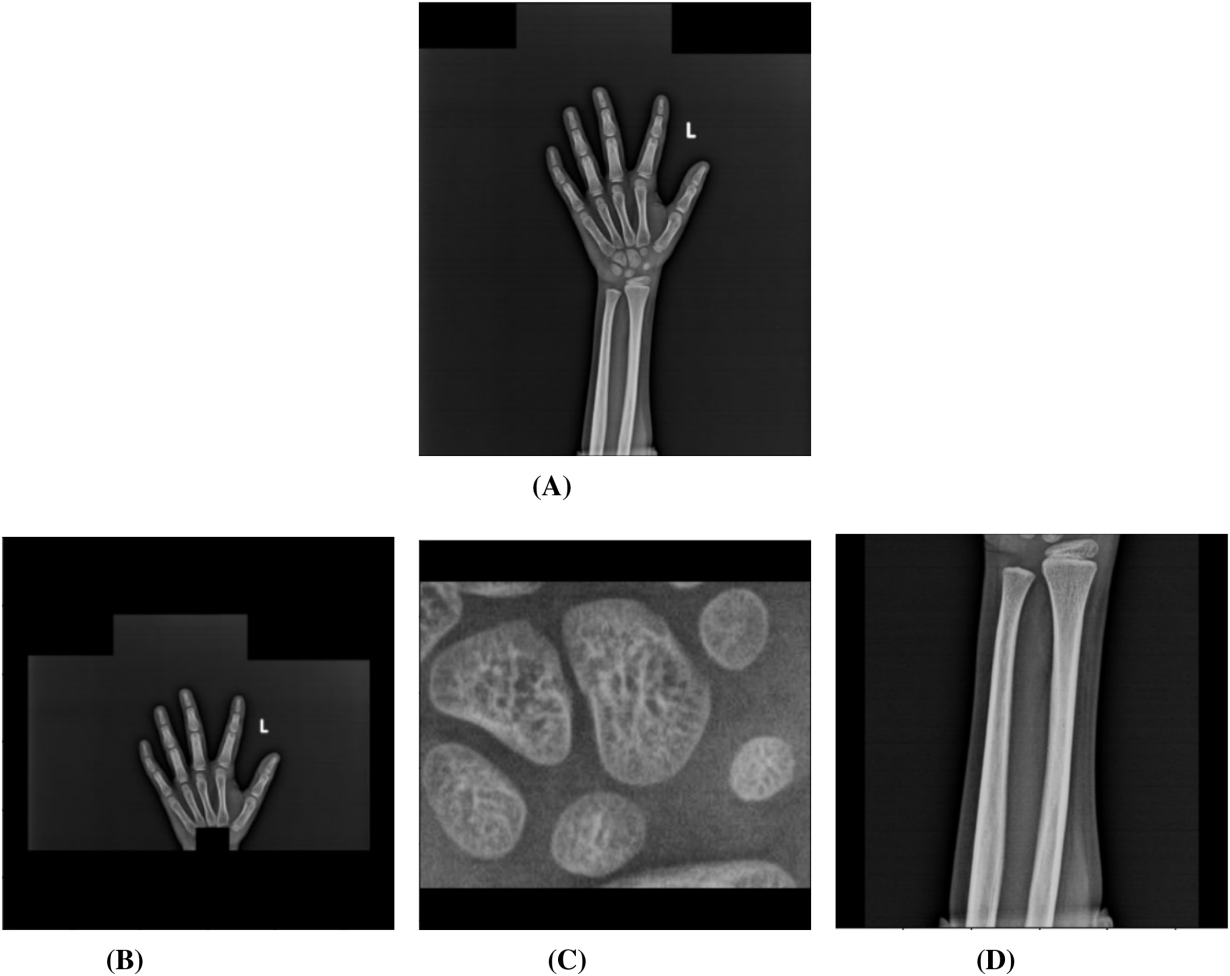
The full-hand image **(A)** was successfully segmented into Shortbones **(B)**, Carpals **(C)** and Wrist **(D)** after applying various mask post-processing techniques such as morphological operations, histogram equalization and area- and intensity-based appropriate contour-detection.

These generated masks were further refined to remove noise using various mask post-processing methods, such as morphological operations, precise contour detection through area and mean-pixel value thresholding to obtain the three segments (**Figure 1**). The detailed steps of mask refinement, along with example images of intermediate and final segmentation masks, are included in the **Supplementary Material**, Section 1.

### 2.4 CNN architecture

This study used the DenseNet161 architecture framework for predicting bone age. The efficient feature map reuse and its dense connectivity pattern make DenseNet a good candidate architecture for medical image analysis.

From a practical standpoint, DenseNet161 represents a well-established and widely used backbone in medical imaging. Our primary objective was to investigate whether biologically informed segmental modeling, when combined with population-specific fine-tuning, improves bone age estimation—rather than to optimize architectural novelty. Importantly, this work focuses on adapting an RSNA-trained model to Indian pediatric data through targeted fine-tuning. In this context, using a legacy backbone, previously describe in Chapke (32) and Chapke et al. (33), served as a controlled and reproducible feature extractor, enabling us to demonstrate that meaningful performance improvements can be achieved by aligning learned representations with Indian skeletal maturation patterns.

### 2.5 Base model training on RSNA data

Chapke (32) transfer learned pretrained Densenet-161 model to the RSNA dataset for bone age assessment. They trained the three segment models independently against their GP ground truths and found that the MAD indicated good generalization capability of the model to the RSNA dataset (see **Table 2**).

**Table 2:** Mean absolute difference (MAD) in months for different segments and models.

Segment	Model	Training data	Validation data	Male MAD (months)	Female MAD (months)
Full-Hand	Base-Model	RSNA	RSNA	7.2	8.4
Full-Hand	Base-Model	RSNA	HCJBA	12.4	9.4
Full-Hand	Transfer learned-model	HCJBA	HCJBA	5.7	5.9
Short-bones	Base-Model	RSNA	RSNA	7.4	7.7
Short-bones	Base-Model	RSNA	HCJBA	38.1	23.4
Short-bones	Transfer learned-model	HCJBA	HCJBA	8.3	8.6
Carpals	Base-Model	RSNA	RSNA	8.6	9.3
Carpals	Base-Model	RSNA	HCJBA	14.8	19.5
Carpals	Transfer learned-model	HCJBA	HCJBA	9.6	9.2
Wrist	Base-Model	RSNA	RSNA	8.8	9.7
Wrist	Base-Model	RSNA	HCJBA	15.9	10.1
Wrist	Transfer learned-model	HCJBA	HCJBA	6.8	7.5
Combined	SGP model	HCJBA	HCJBA	4.7	4.9

For the HCJBA dataset, the training and validation sets comprised 1,071 and 179 male X-rays, and 1,011 and 169 female X-rays, respectively. Training and validation dataset sizes for the RSNA were n=6,833 and n=773 for males, and 5,778 and 652 for females, respectively.

### 2.6 Same-domain transfer learning on HCJBA dataset

The base RSNA trained model demonstrated poor generalization when tested on the HCJBA dataset. This performance gap could be rooted in the ethnicity difference, labeling biases or imaging protocols between the two datasets. To address this issue, we adopted a same-domain transfer learning strategy, which leverages the inherent similarity between the source (RSNA) and target (HCJBA) domains. This approach allows the model to retain domain-relevant low-level representations learned during pre-training while fine-tuning to the statistical characteristics of the target dataset (35). It prevents overfitting the model for cases where labeled data is limited.

For fine-tuning, all layers of the RSNA-trained base model were frozen except the final layer. This final layer was retrained using normalized images from the HCJBA dataset and their corresponding ground-truth labels, which were obtained by averaging the bone age ratings provided independently by two experienced clinicians. Normalization was performed using the RSNA dataset-derived mean and standard deviation to preserve consistency with the pretrained feature distributions. Subsequently, only the final prediction layer was fine-tuned using HCJBA labels, allowing the model to adapt the learned feature representations to the bone age distribution of the Indian cohort. The segmental models were fine-tuned against their respective segmental bone age ground truth. Separate full-hand and segmental models were trained for each of the male and female subsets of the entire dataset to account for sex-specific differences in bone maturation. The models’ performance was evaluated using MAD. Correlation and Bland-Altman analysis were used to quantify the level of agreement between the ground truth and the models’ predictions. The hyperparameter configurations used for all models are summarized in the **Supplementary Material** (Section 2).

### 2.7 The segmental Greulich-Pyle age

Segmental maturity is non-uniform across different regions of the hand. Therefore, independent segmental models can have different predictions, each exhibiting a specific aspect of bone age maturation. To obtain a clinically coherent estimate of bone age that accounts for this inter-segmental variability, we combine the average of the three segmental predictions (*Seg_Avg*) with the full-hand prediction (*fh*) using a convex combination, that is, a combined prediction $C_{i}$ (Equation 1):

(1) $$C_{i}=\alpha Seg\_Avg_{i}+\left(1-\alpha \right)fh_{i},$$

where each $C_{i}$ is then mapped to the closest GP age: ${\tilde{C}}_{i}=\text{arg }\min_{g\in G}\left|C_{i}-g\right|,\;$where G denotes the set of discrete GP classes. An optimal $\alpha^{*}$ (Equation 2) is obtained by minimizing the L2 norm between the predicted ${\tilde{C}}_{i}$ age and the full-hand ground truth $y_{i}$ over all the training samples. Specifically, α was swept over the interval [0,1], and the L2 error was computed on the validation set for each candidate value. The value of α that minimized this error was selected independently for the male and female cohorts. Formally, this optimization is expressed as:

(2) $$\alpha^{*}=\text{arg }\underset{\alpha }{\min}{∥{\tilde{C}}_{i}-y_{i}∥}_{2}^{2}.$$

For inference, we use the optimal *α*^∗^ to compute the *Segmental Greulich-Pyle (SGP) age* (Equation 3) for each sample,

(3) $$SGP_{i}=\alpha^{*}Seg\_Avg_{i}+\left(1-\alpha^{*}\right)fh_{i},$$

once this is aligned to the closest GP class.

This method assigns a unified bone age rating to the child, given its corresponding full-hand and segmental bone ages. The SGP age is compared to the full-hand ground truth *y* to calculate the MAD and its level of agreement with the reference ratings.

## 3 Results

### 3.1 Base and transfer learned model’s performance on predicting bone age for full-hand images

**Figure 2:**
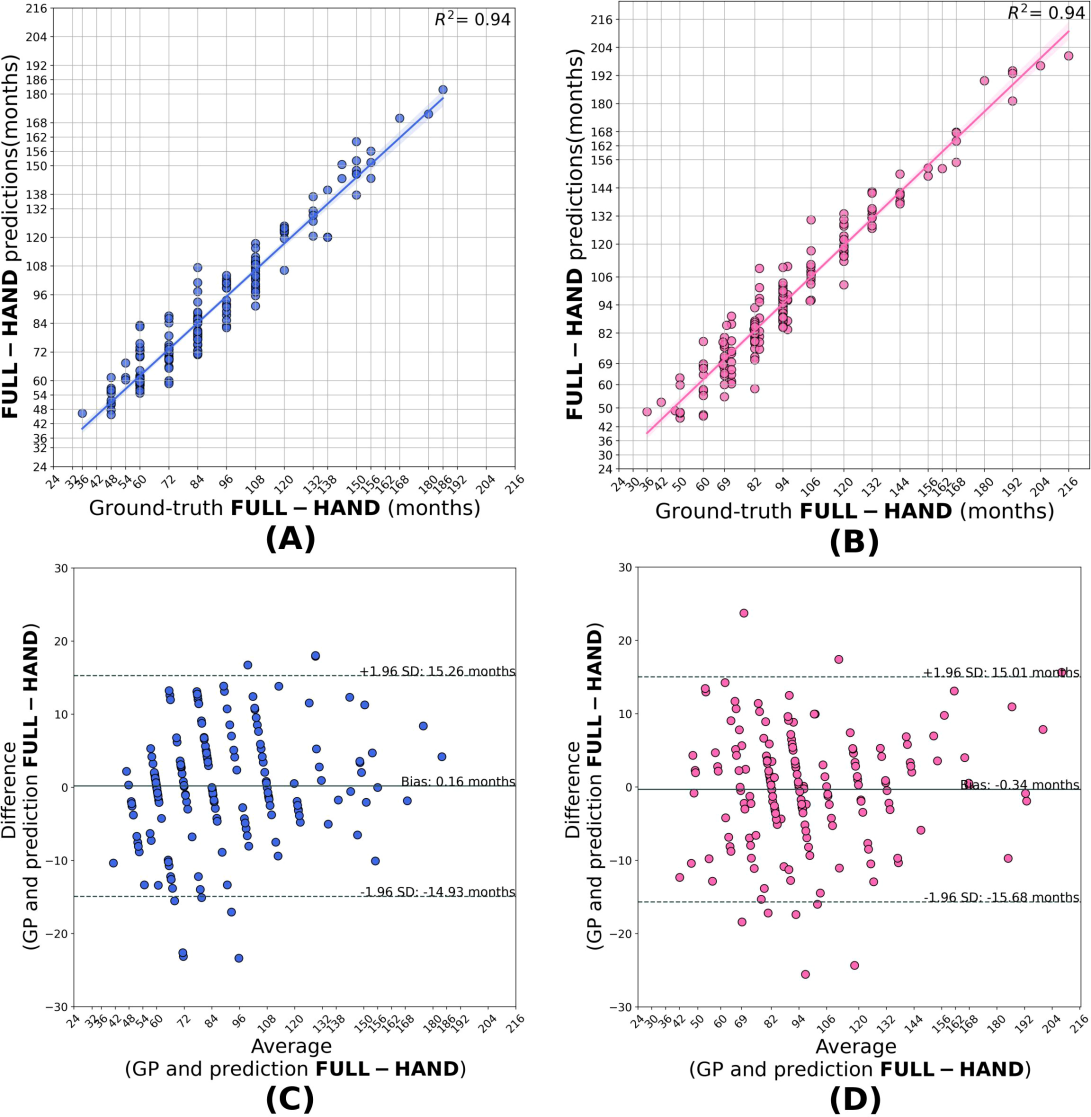
Correlation **(A, B)** and Bland–Altman **(C, D)** plots for the full-hand model predictions in males and females. The model predictions shows good agreement with the full-hand ground truth with an *R*^2^ = 0.94 for male and *R*^2^ = 0.94 for girls, with biases of +0.16 and −0.34 months, respectively.

Same-domain transfer learned full-hand models outperformed the base model in predicting the full-hand and segmental bone ages, as evident from the lower MAD values compared to base model (see **Table 2**). Hence, the transfer-learned models effectively mitigated the domain shift, potentially caused by the out-of-distribution variations (see **Supplementary Material**, Section 3). The predictions of the full-hand model showed a strong correlation with the ratings provided by the clinicians (*r*^2^: 0.93 for males and 0.94 for females). The Bland-Altman plot shows a high level of agreement between the model’s prediction and the expert ratings with biases of 0.16 months for males and -0.34 months for females (**Figure 2**). Predictions lying beyond ±2 standard deviations from the mean difference were considered high-error cases, and representative examples of these radiographs are provided in the **Supplementary Material**, Section 5.

### 3.2 Base and transfer learned model’s performance on predicting bone age for segmental images

**Figure 3:**
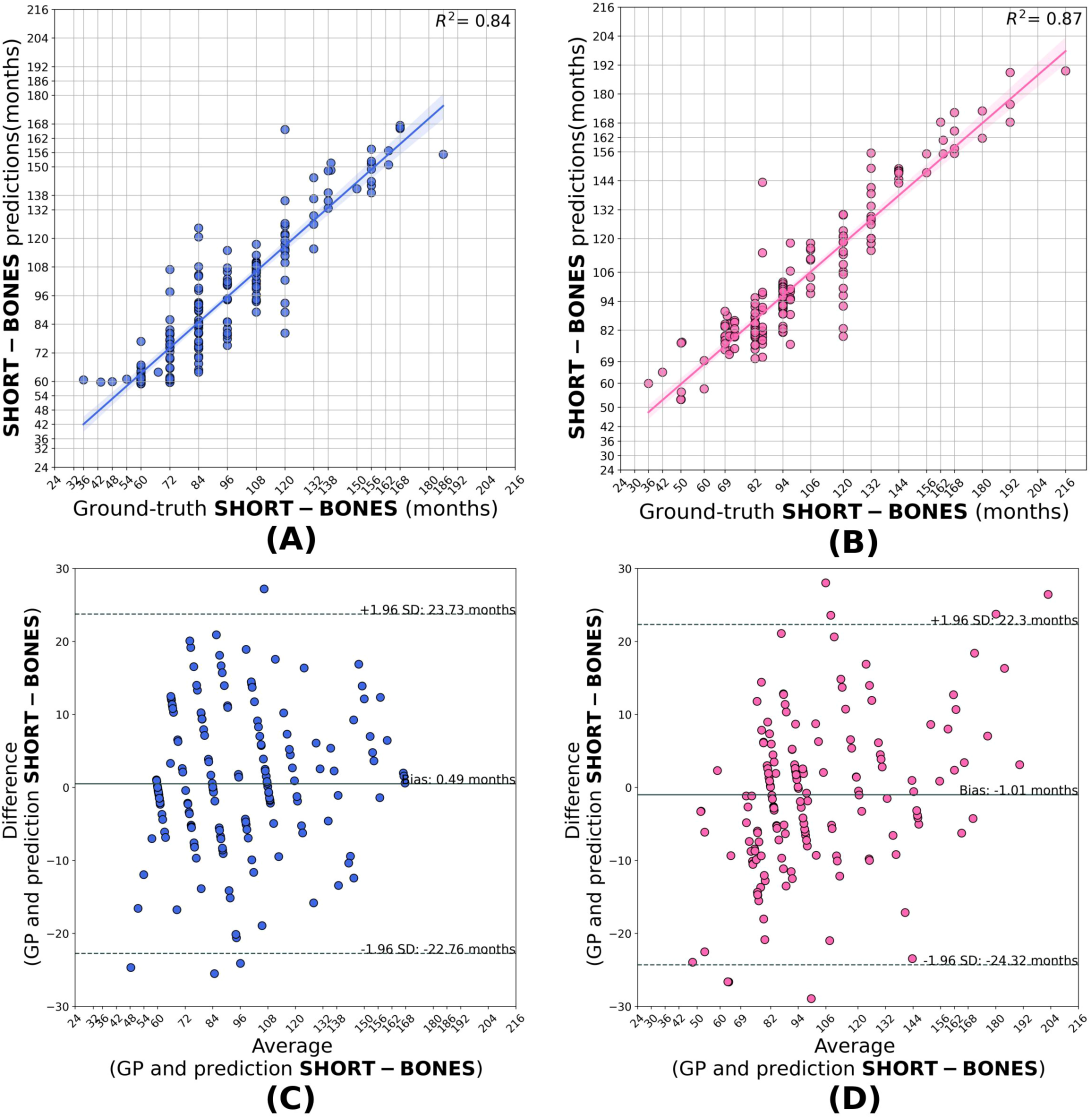
Correlation **(A, B)** and Bland–Altman **(C, D)** plots for the short-bone model predictions in males and females. The model shows strong agreement with the ground truth, achieving *R*^2^ = 0.84 for males and *R*^2^ = 0.87 for females, with biases of +0.49 and −1.01 months, respectively, indicating a slight overestimation for females.

**Figure 4:**
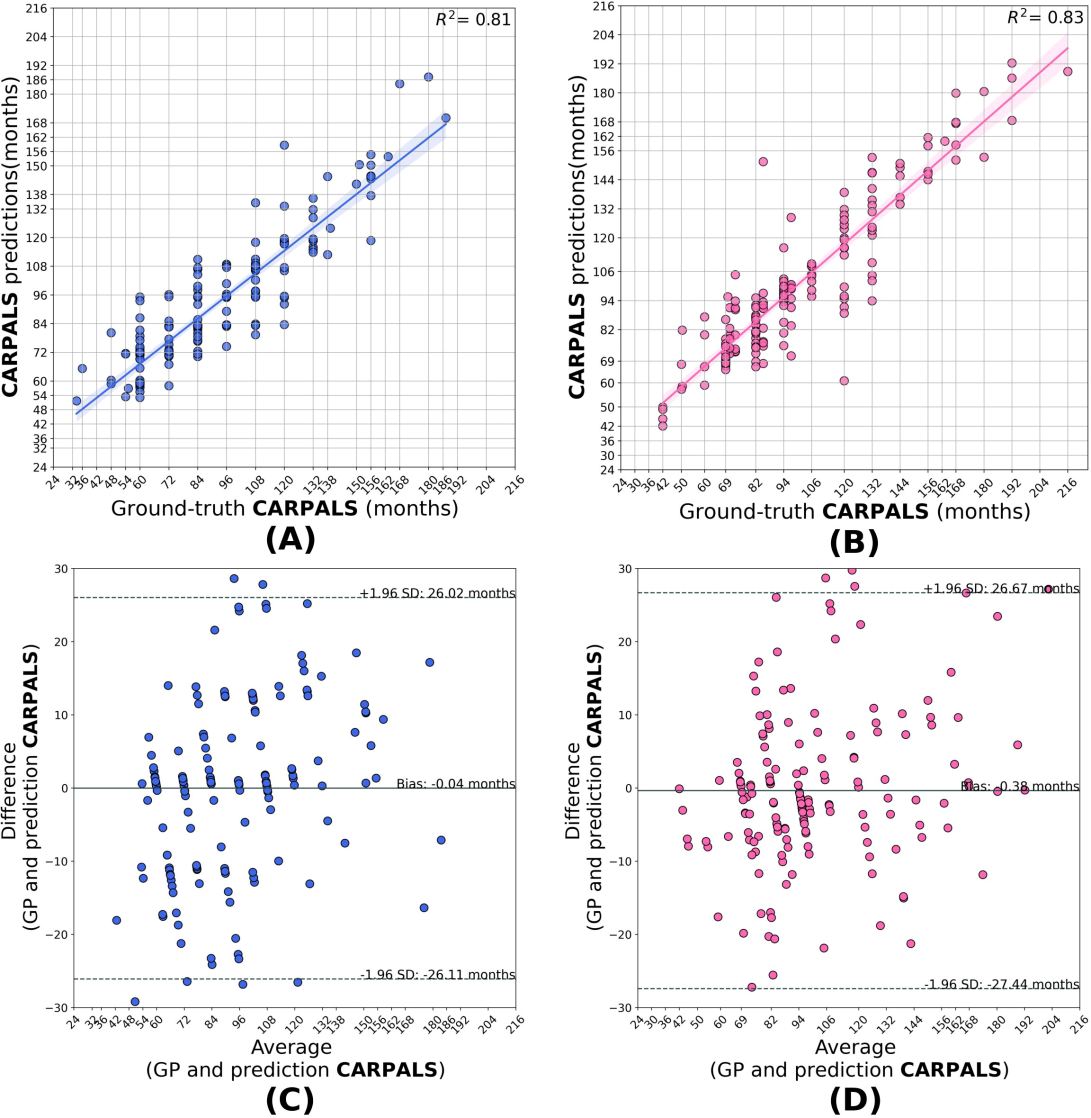
Correlation **(A, B)** and Bland–Altman **(C, D)** plots for the carpal model predictions in males and females. The model attains *R*^2^ = 0.81 for males and *R*^2^ = 0.83 for females, with biases of −0.04 and +0.38 months, respectively. Note that the carpal model shows negligible systematic deviation (bias) from the ground truth in both sexes.

**Figure 5:**
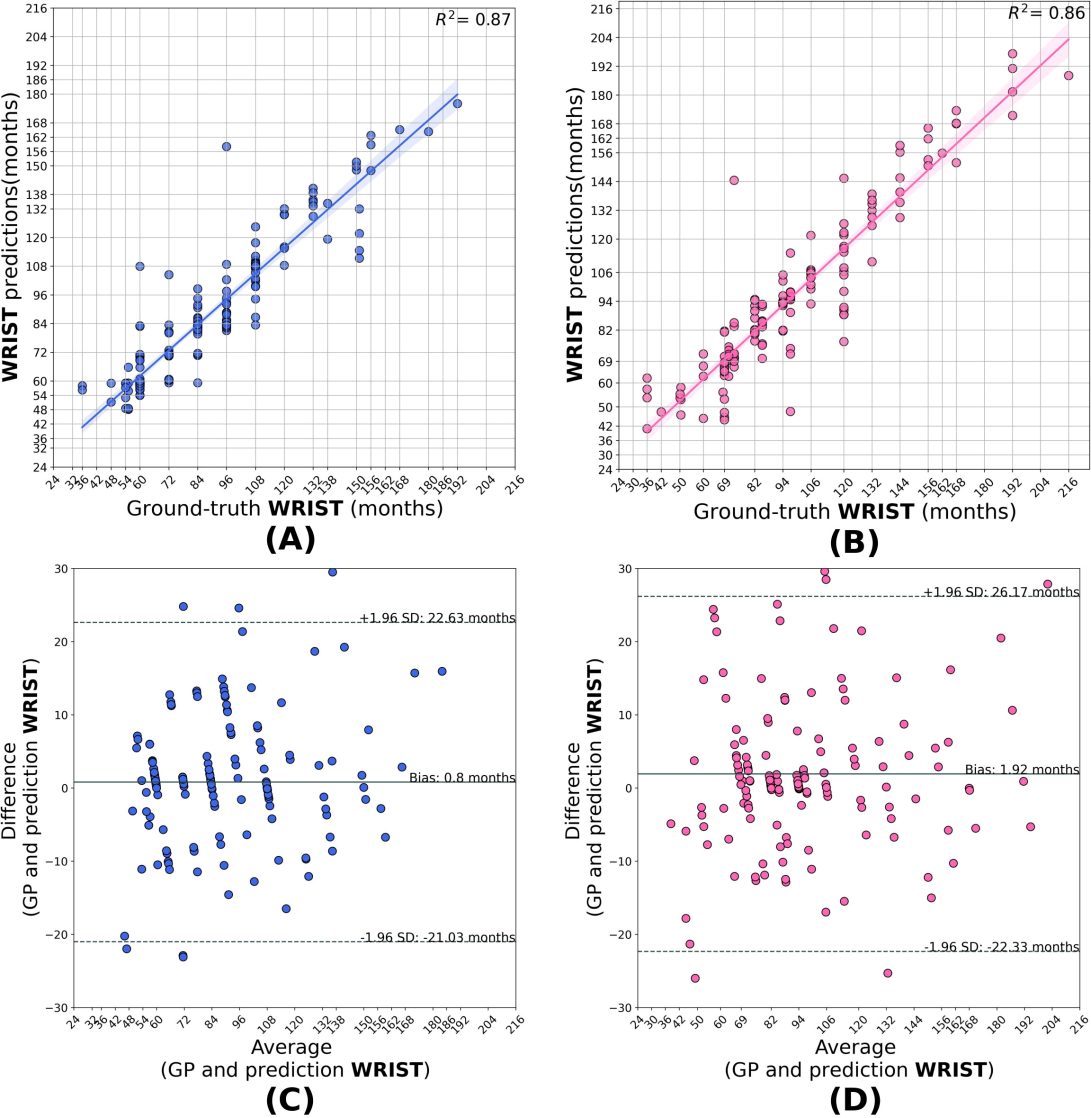
Correlation **(A, B)** and Bland–Altman **(C, D)** plots for the wrist model predictions in males and females. The model attains *R*^2^ = 0.87 for males and *R*^2^ = 0.86 for females, with biases of +0.80 and +1.92 months, respectively, showing a mild underestimation trend.

Compared to the base model, the transfer learned segment models generalized better when applied to the HCJBA dataset. Male and female *r*^2^ values for the short bones model were 0.84 and 0.87, respectively. The Bland-Altman analysis showed a bias of -1.01 months for females and 0.49 months for males, suggesting a slight overestimation for females. With Bland-Altman biases of -0.04 months and 0.38 months, respectively, the carpal models produced *r*^2^ values of 0.81 for males and 0.83 for females, showing negligible systematic deviation from the ground truth. With *r*^2^ values of 0.87 for males and 0.86 for females, the wrist models performed the best overall. A slight underestimation trend was suggested by the corresponding biases, which were 0.80 and 1.92 months, respectively (**Figures 3**–**5**).

However, the segmental models consistently showed inferior performance than the full-hand model (**Table 2**). When training on isolated segments of the hand, the model loses contextual anatomical information, potentially limiting the model from performing as well as the full-hand model. Among the segment models, the carpal-only model exhibited the highest MAD value. This pattern is consistent with the base segmental prediction models.

### 3.3 Base and transfer learned model’s performance on predicting bone age by SGP method

**Figure 6:**
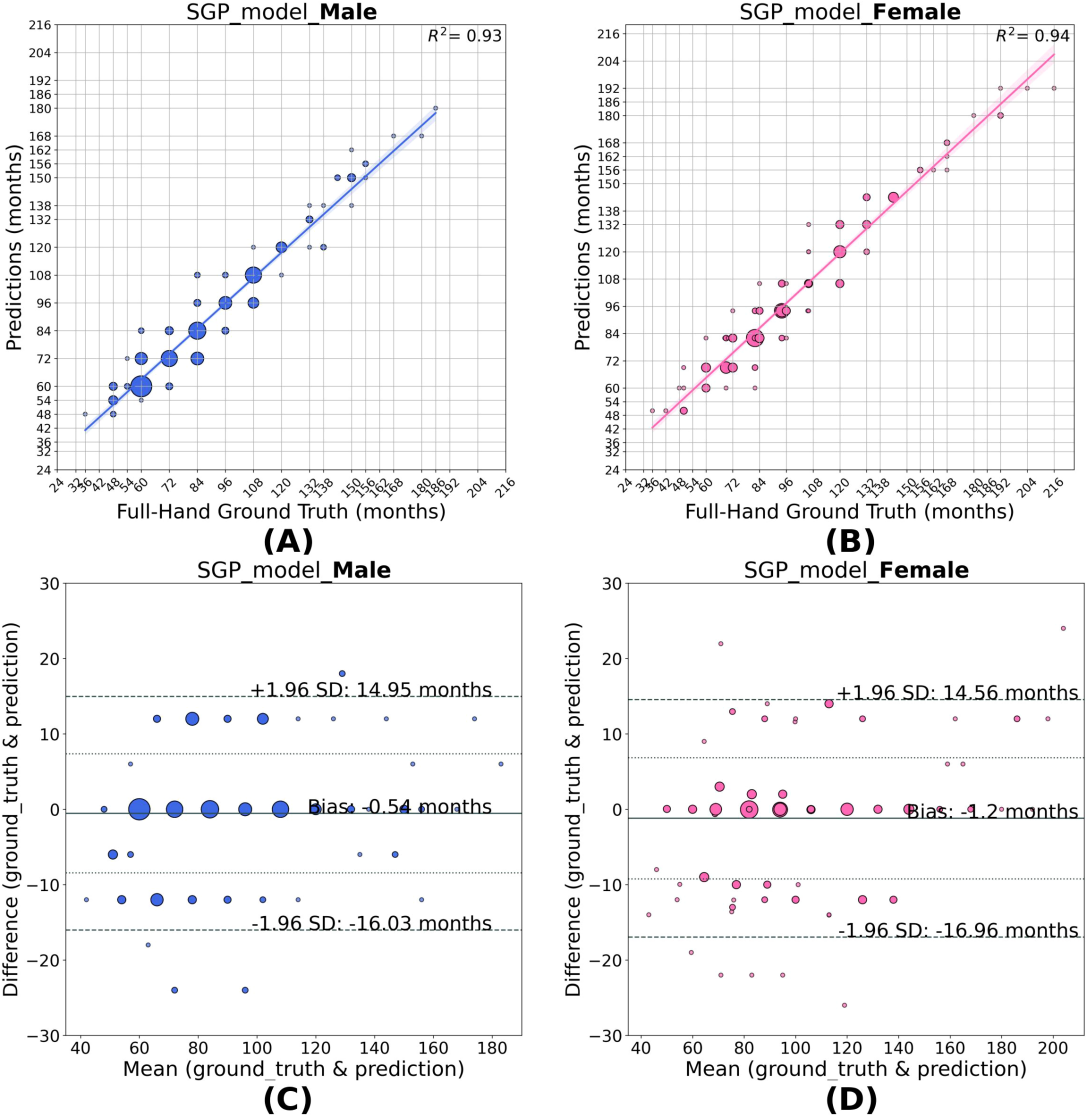
Correlation **(A, B)** and Bland–Altman **(C, D)** plots for SGP model predictions in males and females. As the SGP model outputs discrete GP classes, the size of each scatter point represents the frequency of predictions corresponding to a given GP class for a specific full-hand ground truth. The model predictions show good agreement with the full-hand ground truth with an *R*^2^ = 0.93 for males and *R*^2^ = 0.94 for females, with biases of −0.54 and −1.20 months, respectively, a slight overestimation for females.

The SGP predictions outperformed the full-hand model, underscoring the fact that integrating the information of segmental variations improves bone age estimation. The SGP model achieved MAD values of 4.75 months for the males and 4.93 months for the females. The female SGP model equally weighs the segmental average and the full-hand prediction with an *α* value of 0.49. On the contrary, the male SGP model puts more weight on the full-hand predictions than the segmental average, with an *α* value of 0.24. The optimization landscape of *α* for male and female model is illustrated in **Supplementary Material**, Section 4. The agreement of the SGP method with the ground truth labels is illustrated in the correlation and Bland-Altman plots (**Figure 6**). The SGP model predictions showed high correlation with the full-hand ground truth values by achieving an *r*^2^ value of 0.93 for males and 0.94 for females. The Bland-Altman analysis showed a mean bias of -0.54 months for males. It slightly underestimates the SGP age for females by 1.2 months when compared to full-hand ground truth.

## 4 Discussion

This work introduces digiBONE, the first automated BAA system to systematically incorporate clinically relevant segmental maturity patterns into a deep learning framework. The proposed Segmental Greulich–Pyle (SGP) method provides a segmental perspective of skeletal development, which is overlooked in the traditional GP method, enabling interpretable understanding of bone maturation across different anatomical regions of the hand. Additionally, the system can be integrated into fast-paced clinical workflows since it provides predictions in less than five seconds. Our results indicate that using a biologically informed approach achieves a more appropriate and personalized bone age estimate than models that rely solely on full-hand-only images, demonstrating its potential for interpretable and precise pediatric skeletal maturity assessment.

The automation of BAA using computer vision techniques offers a scalable solution to well-known challenges in manual assessment, including observer variability and time constraints. Deep learning methods—particularly convolutional neural networks (CNNs)—are well-suited to this problem because of their ability to extract hierarchical image features and iteratively learn complex non-linear mappings from radiographs to developmental age. Beyond population alignment, traditional GP-based BAA methods, whether manual or automated, typically treat the hand as a single anatomical unit. However, clinical literature (1, 31) indicates that skeletal development is non-uniform across all hand regions—metacarpals, phalanges, carpals, and wrists mature at different rates due to various hormonal influences. We observed that our *full-hand* CNN models generally produced predictions that deviated by at most one GP class from the ground truth. While part of this variation can be attributed to statistical prediction error, another plausible explanation is that the models detect subtle maturity differences within the same GP class —differences that are not explicitly captured in the class-aligned ground truth. This aligns with our hypothesis that segmental maturity differences influence prediction spread. A full-hand CNN model derives global morphological cues, while models trained on isolated anatomical segments specialize in local feature patterns. This is not just a spatial difference; it represents fundamentally different feature spaces and, importantly, different target variables. For example, a given radiograph may have its respective full-hand GP-class label, yet individual segments could demonstrate developmental stages that is not consistent with this global classification. In essence, the four independent models are learning complementary “truths” about the same X-ray image. digiBONE models this heterogeneity by learning differential maturation patterns across anatomically and biologically coherent hand segments, in contrast to TW3-based approaches, which quantify skeletal maturity through independent bone-level scores. Segmental models have less global morphological information, and thus generally yield higher MAD values when used alone versus the full-hand models. Among these, the carpal model showed the highest MAD. This can be attributed to the biological maturation of the carpal bones: following the average age of onset of puberty in the Indian population [11.5 years for males and 10.5 years for females (31)], carpal bones reach full maturation. Hence, the loss of discriminative power of carpal images for post-puberty GP classes might be weakening the mapping of carpal images to their labels while training, leading to a poorer performance of the carpal-only model compared to the other models. Importantly, the model does not explicitly learn to down-weight carpal information as a function of age. Incorporating an age-adaptive weighting mechanism could be a possible future direction which would be more closely aligned with clinical practice. However, the model that resulted from combining segmental and full-hand predictions using the SGP framework had the lowest overall MAD (see **Table 2**). The MAD for males and females using the full-hand-only model were 5.7 and 5.9 months, respectively. MAD value decreased to 4.7 months for males and 4.9 months for females when segmental averages were taken into account. These results clearly highlight that incorporating segmental information refines bone age estimation better than what was achieved with global hand features alone.

The key observation, however, lies in the optimal weighting strategy. Equally weighting the full-hand and average segmental predictions produced the lowest error for females. In contrast, for males, empirically, only around 20% weight was assigned to average segmental predictions, with the remaining 80% derived from full-hand predictions. This suggests that segmental models contribute differently to prediction accuracy depending on sex. The observed difference underscores the importance of sex-specific weighting strategies in automated bone age assessment. Future work will consider a more sophisticated weighting or ensemble mechanism—potentially learned from data—that could further optimize integration performance.

### 4.1 Population-to-individual level assessment

Our approach trained models on large-scale population data to capture class-specific skeletal patterns. In addition, we developed multiple segmental models, each emphasizing different aspects of the radiograph by focusing on distinct anatomical segments. When applied to individual radiographs, these population-trained models generate predictions that reflect how a given patient aligns with the broader population trends. The process of reconciling predictions from different models gives rise to personalization.

Population to individual level assessment leverages the segmental skeletal maturation. Two radiographs may be assigned the same bone age class under the Greulich-Pyle system, yet differ meaningfully at the segmental level—for example, one child may exhibit advanced maturation of tubular bones while another shows more advanced carpal development. Such differences are not captured from the full-hand model but are clinically relevant at the individual level. Our approach uses segmental models to complement full-hand predictions to capture these clinical differences. While full-hand model group radiographs into GP classes, segmental predictions reveal where two children of the same “bone age” may differ biologically. This patient-specific insight improves the interpretability of automated predictions and explains the heterogeneity of the classes. It improves personalization by going beyond a single global prediction to a thorough understanding of the underlying developmental truth at the individual level.

In summary, digiBONE demonstrates that incorporating segmental maturation patterns into deep learning substantially improves accuracy and interpretability over full-hand–only approaches. The framework not only addresses known sources of variability, such as asynchronous skeletal maturation, but also advances automated BAA toward personalization by bridging population-level learning with individual-level maturation profiles. While the present work focuses on validating the proposed segmental strategy using a well-established CNN backbone, future studies will explore newer architectures. Finally, it is interesting to ask how widely can the current model be applied to various Indian cohorts. We hope to conduct multi-center studies which can independently validate the models further in the future.

## Data Availability

The raw data supporting the conclusions of this article will be made available by the authors, without undue reservation. The RSNA Pediatric Bone Age Challenge dataset used in this study is publicly available for academic research and non-commercial purposes under the terms and conditions defined by the Radiological Society of North America (RSNA) (9). The dataset is de-identified and can be accessed at: https://www.rsna.org/education/ai-resources-and-tools/ai-image-challenge/rsna-pediatric-bone-age-challenge-2017. The segmentation model, along with the base model and transfer-learned models for bone age estimation, is publicly available on GitHub at the following link: https://github.com/goellab/digiBONE.
