# Supplementary material for: digiBONE: an automated tool for segmental Greulich-Pyle bone age assessment of Indian children and adolescents
Source: Front Endocrinol (Lausanne). 2026 Mar 11;17:1757571. doi: 10.3389/fendo.2026.1757571 (PMC13012973; doi:10.3389/fendo.2026.1757571)
Supplement: Supplementary file 1 [file DataSheet1.pdf]

Supplementary Material

1 SEGMENTATION MASK POST-PROCESSING

Chapke (2023) developed X-ray image segmentation models using the u-net architecture, trained on the RSNA data set. Independent models were trained for short bones, carpals and radius-ulna, with each producing masks that delineated the corresponding regions of interest (ROIs). These masks were subsequently processed with the OpenCV library which generated bounding boxes around these masks using contour detection, enabling precise segmentation of the anatomical structures. (Figure 1).

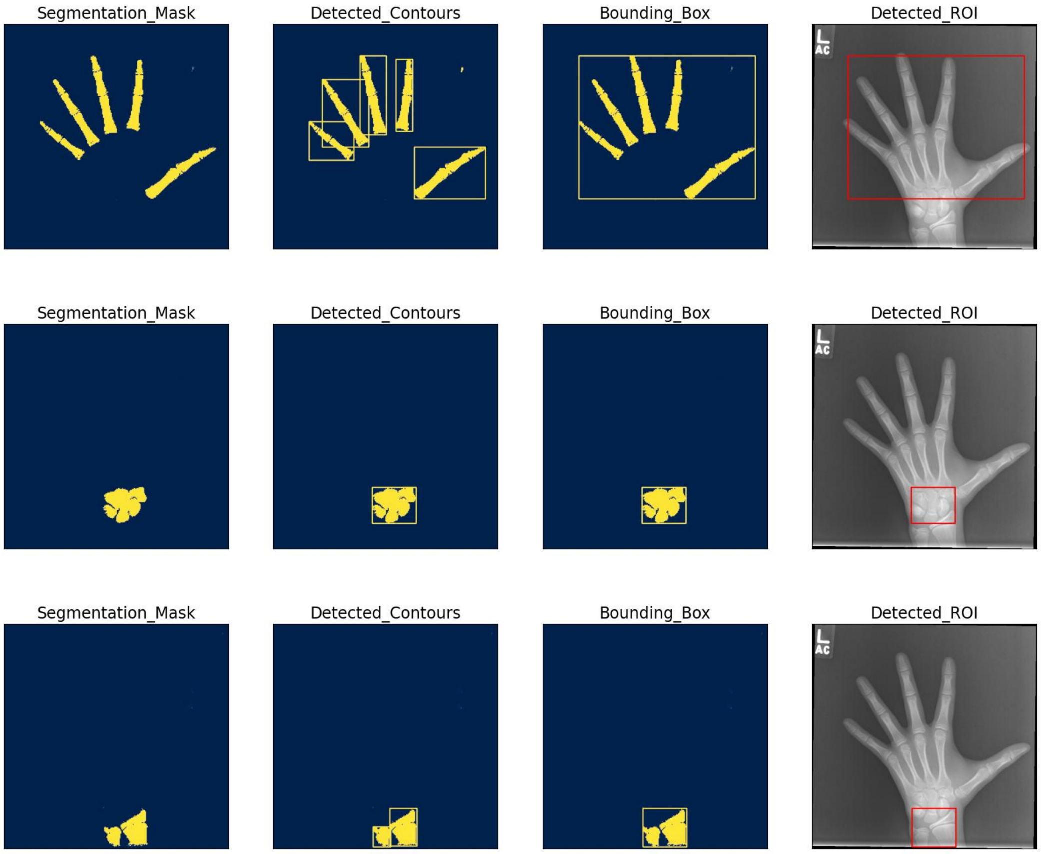

Figure 1. Segmentation model outputs without mask post-processing on RSNA hand X-ray images for short-bones, carpals, and wrist regions (from top to bottom). Each row corresponds to the intermediate stages of ROI detection— segmentation mask prediction, contour detection of the generated masks, bounding box generation based on detected contours, and the corresponding isolation of the ROI from the full-hand image. The model effectively identified the target regions and generated appropriate masks, enabling accurate ROI localisation for subsequent age prediction.

When applied to the HCJBA dataset, however, the segmentation models produced masks with considerable noise, which hindered the accurate delineation of ROIs. Hence, discovering the exact contour of the region of interest was impossible. This discrepancy made direct extraction of precise contours infeasible, necessitating the use of mask post-processing techniques. (Figure 2).

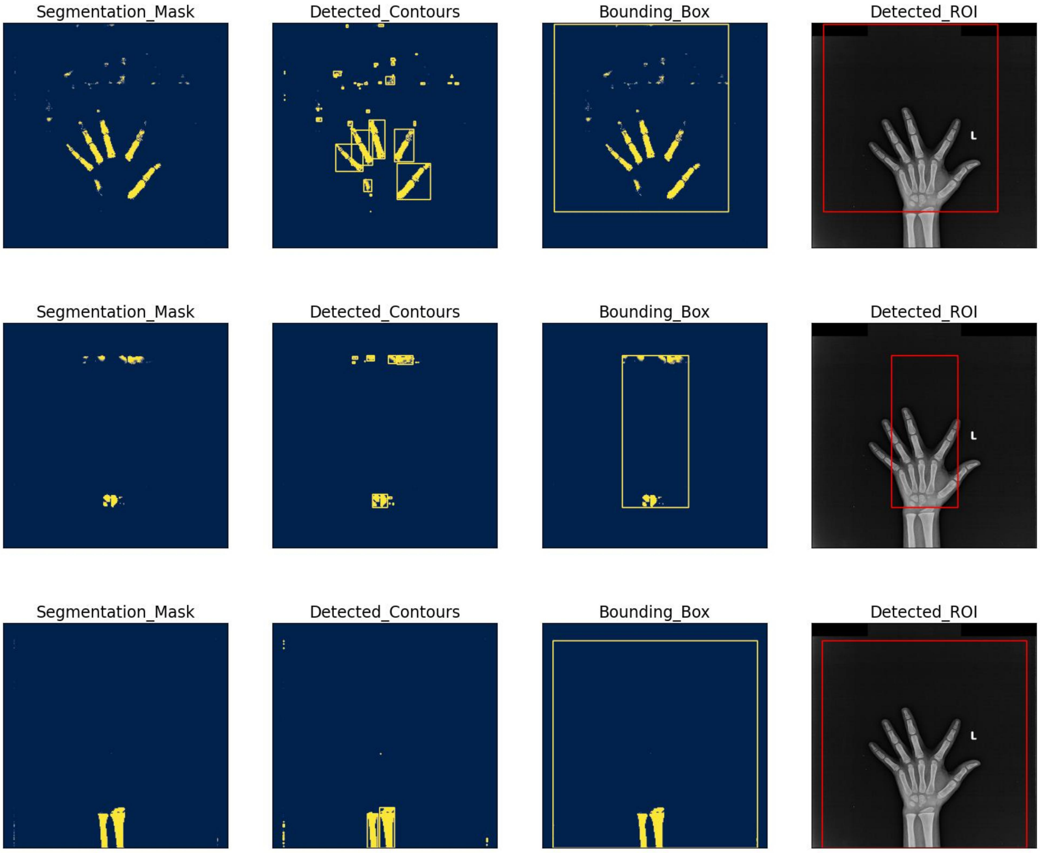

Figure 2. Segmentation model outputs without mask post-processing on HCJBA hand X-ray images for short-bones, carpals, and wrist regions (from top to bottom). Each row corresponds to the intermediate stages of ROI detection— segmentation mask prediction, contour detection of the generated masks, bounding box generation based on detected contours, and the corresponding isolation of the ROI from the full-hand image. The model generated noisy masks, that hindered the accurate ROI localisation of the HCJBA dataset for subsequent age prediction.

The post-processing steps involved morphological operations and histogram equalisation. Morphological operations like dilation or erosion add or remove pixels respectively from the object edges of an image. Histogram equalisation, applied as a preprocessing step, is an intensity transformation technique that enhances the image's contrast. It uniformly distributes the intensity in the resultant image and brings more image details to light facilitating more accurate ROI identification (Figure 3). Morphological operations were subsequently employed on the generated masks to suppress residual noise. Specifically, erosion was applied to eliminate small spurious regions, followed by dilation to restore the structural integrity of the segmented contours.

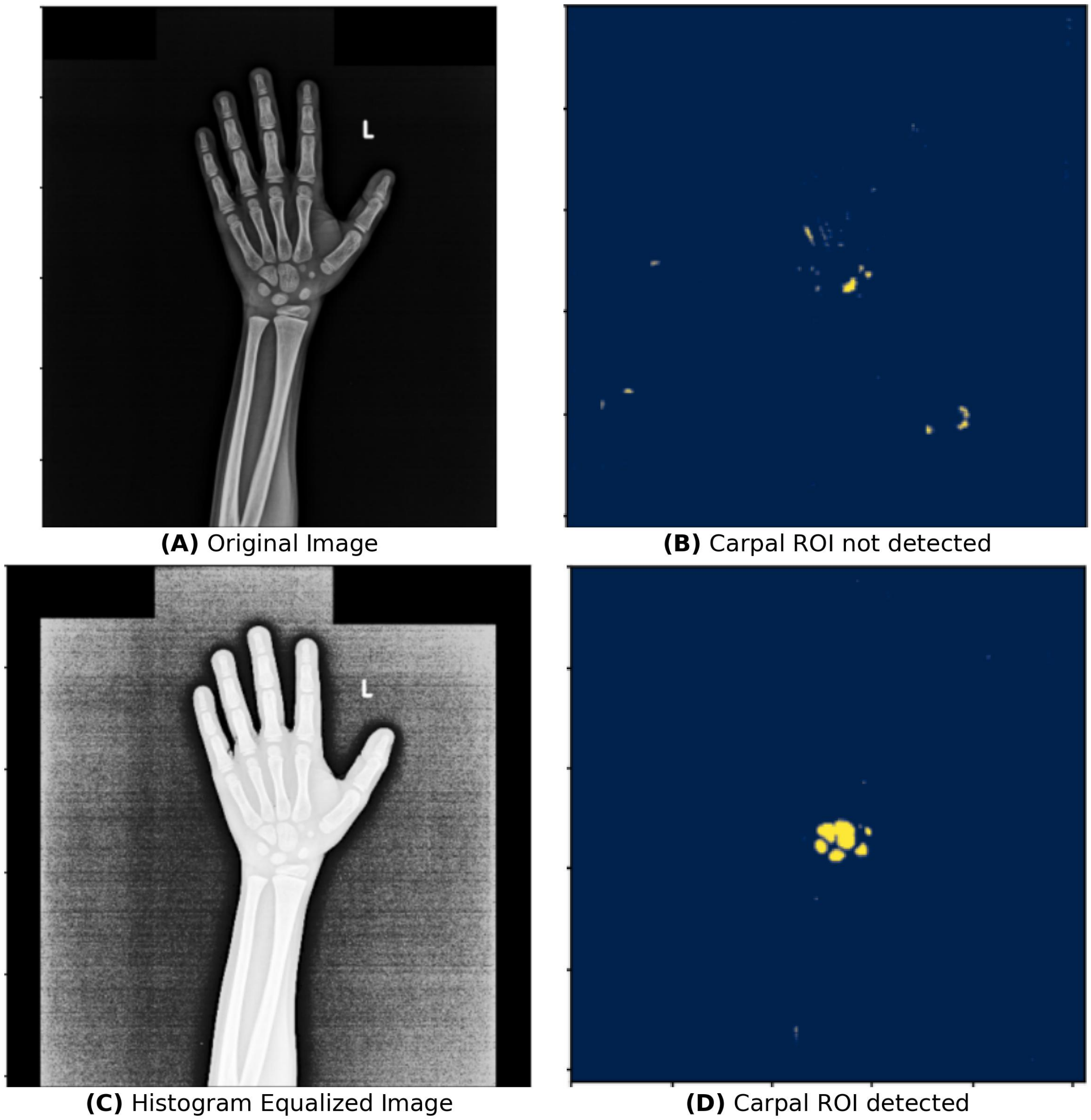

Figure 3. Identification of Carpal ROI in HCJBA dataset. Histogram Equalization enhanced the contrast of the image **(C)** by uniform intensity distribution which led to appropriate Carpal mask generation **(D)** for images in the HCJBA dataset

For the radius–ulna segmentation, the contours corresponding to the radius and ulna were consistently among the largest detected regions, even in the presence of noise. Initially, the segmentation masks for the radius and ulna appeared as distinct entities. Following dilation, however, these masks merged into a single structure, hence the bounding box was generated around the largest contour in the output. This process effectively isolated the radius and ulna as a unified region of interest (Figure 4).

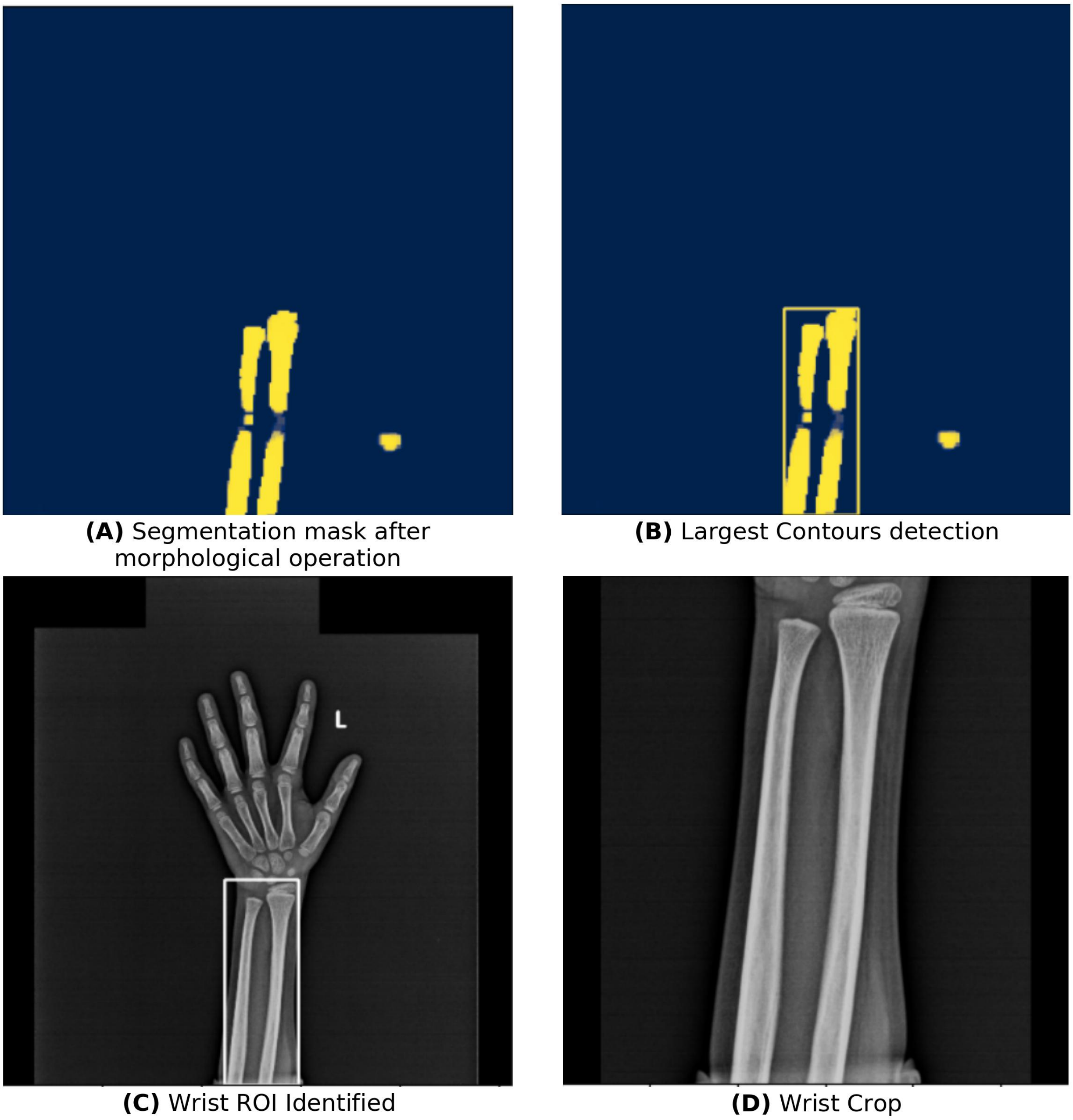

(A) Segmentation mask after morphological operation

(B) Largest Contours detection

(C) Wrist ROI Identified

(D) Wrist Crop

Figure 4. Identification of Wrist ROI in HCJBA dataset. Morphological operations removed noise artifacts and merged the masks of radius and ulna **(A)**. Following this the detection of the largest contour **(B)** led to the identification of the correct wrist ROI **(C-D)** in the HCJBA dataset

A similar contour-based approach was employed for the segmentation of the carpal region. In most cases, identifying and isolating the largest contour successfully produced accurate crops of the carpals. However, the size of the carpal bones varies considerably with age: in younger children, carpals are substantially smaller compared to older children. Empirical observations indicated that, for younger patients, the carpal mask regions frequently matched the dimensions of surrounding noise or, in certain instances, were even smaller. Under such circumstances, simply selecting the

largest contour frequently resulted in the extraction of image background rather than the true carpal region.

Hence, additional intensity-based criterion was added to the process of generating carpal crops to overcome this constraint. In grayscale radiographs, pixel values range from 0 (black) to 255 (white), with the background typically occupying the lowest intensity range near zero. The proposed refinement involved computing the mean pixel intensity of the largest contour. If this value fell below a defined threshold—set at one-quarter of the maximum pixel range (i.e., 64)—the contour was identified as background noise and rejected. The process was then iteratively applied to the next largest contour until a region with a mean intensity above the threshold was identified. This process efficiently differentiated between genuine carpal bone contours and background noise and successfully generated carpal crops (Figure 5).

Once reliable crops for the radius–ulna and carpal regions were obtained, these were subtracted from the original full-hand image, yielding accurate segmentation of the short bones.

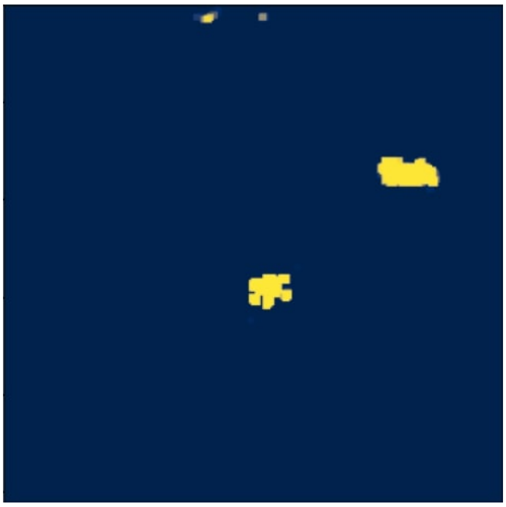

(A) Segmentation mask after morphological operation

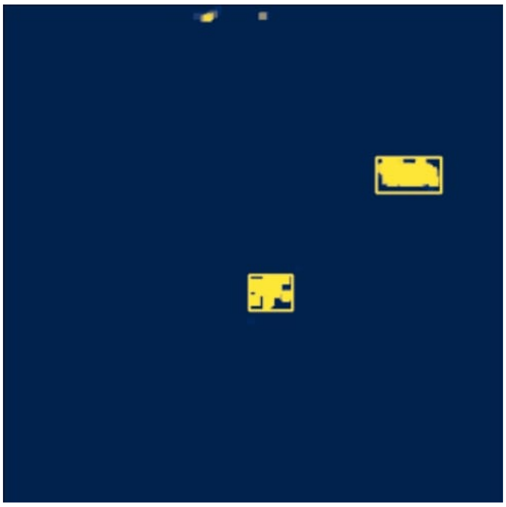

(B) Largest Contours detection

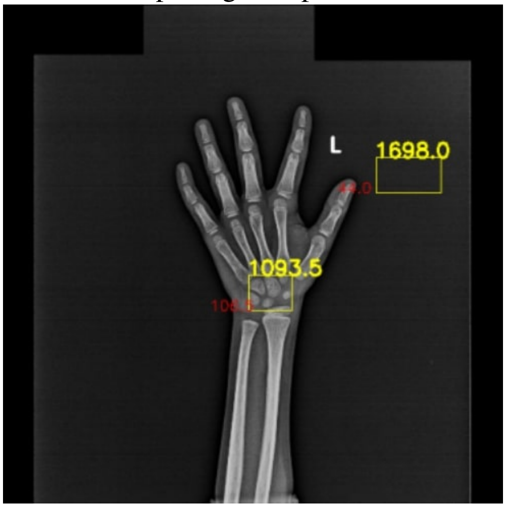

(C) Comparing mean pixel values of detected contours

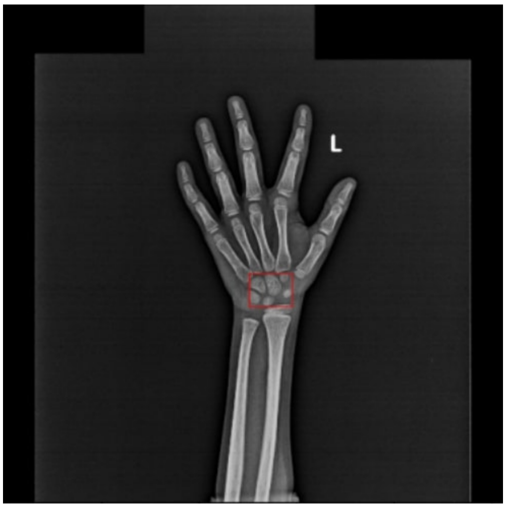

(D) Carpal ROI Identified

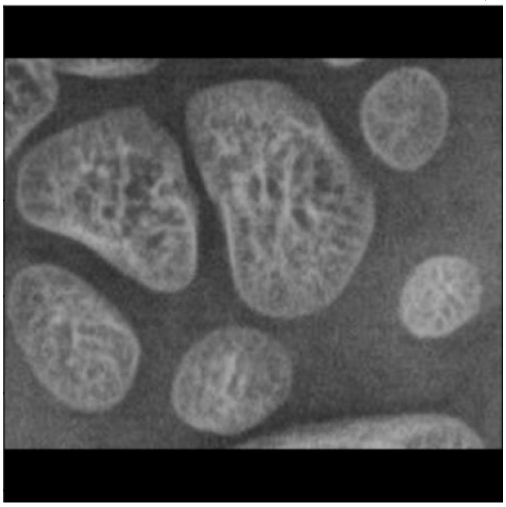

(E) Carpal Crop

Figure 5.

Figure 5. Identifying the carpal region of interest (ROI) from the segmentation mask in HCJBA dataset. Initially, the largest contours were detected to isolate candidate carpal regions **(A-B)**. For younger subjects, where the carpals are smaller and comparable in size to noise, an additional intensity-based criterion was applied by evaluating the mean pixel values of the detected contours **(C)**. The annotations in red represent the mean pixel intensity of each contour, while the annotations in yellow indicates its area. The refined approach accurately identified the true carpal ROI **(D)** and generated the final cropped region **(E)** in the HCJBA dataset.

2 MODEL HYPERPARAMETERS

Hyperparameters were informed by prior RSNA-based bone age assessment studies and refined through limited validation experiments. Exhaustive hyperparameter searches were intentionally avoided due to data constraints and the risk of overfitting, particularly for the HCJBA dataset.

Table 1. Summary of hyperparameters used for training full-hand and segmental models.

Model	Optimizer	Learning Rate	Batch Size
Full-hand (Male)	Adam	3×10^{-4}	32
Full-hand (Female)	Adam	1×10^{-4}	32
Short bones (Male/Female)	Adam	3×10^{-4}	32
Carpal (Male/Female)	Adam	3×10^{-3}	32
Wrist (Male/Female)	Adam	3×10^{-3}	32

3 SYSTEMATIC OVERESTIMATION OF INDIAN BONE AGE BY BASE RSNA MODEL

We compared predictions from the base model trained on the RSNA dataset with the transfer-learned model optimized on Indian data in order to assess the improvement brought about by transfer learning (Figure 6). We observed a clear and similar pattern in the predictions of the models trained separately on male and female data. We see that both the RSNA model (baseline) and the transfer learned model performed well in their respective population. When applied on the Indian dataset, the base RSNA model systematically overestimated the bone age of Indian children. Hence it can be inferred that the transfer-learned model corrected this shift and aligned the Indian dataset closely with its ground truth. This finding suggests that to achieve accurate estimations across different population it is necessary to train population-specific bone age models.

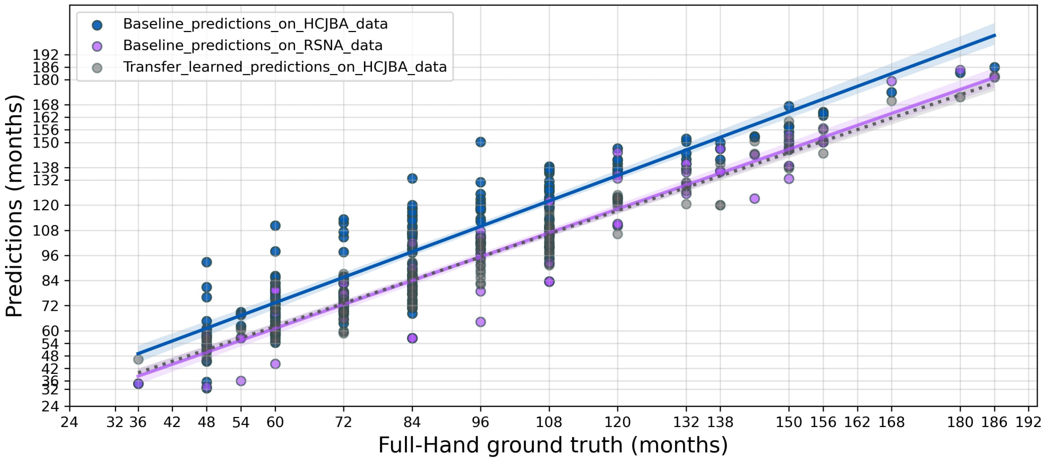

(A) Predictions on Male Dataset

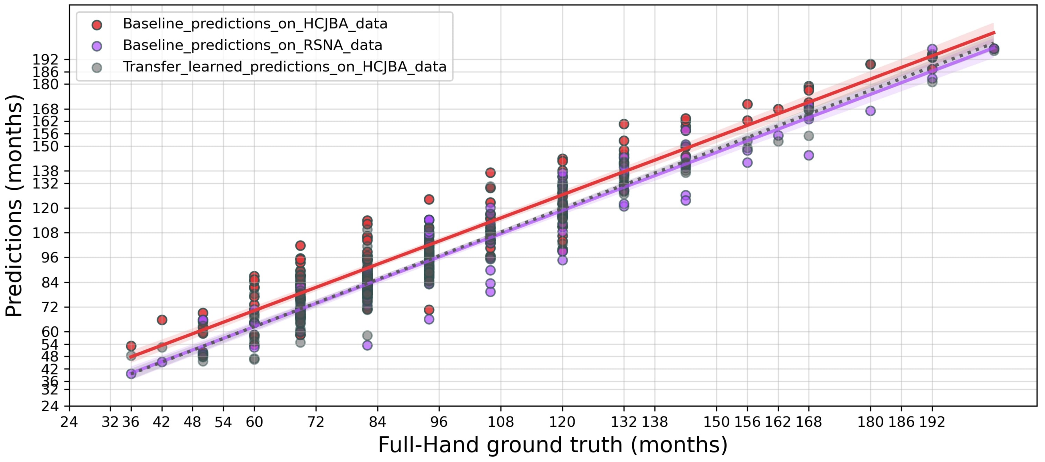

(B) Predictions on Female Dataset

Figure 6. Comparison of bone age predictions from the base RSNA model and the transfer-learned model optimized on Indian data (HCJBA). The RSNA model overestimated bone age for Indian children, whereas the transfer-learned model corrected this bias, aligning predictions more closely with ground truth.

4 SENSITIVITY ANALYSIS OF THE SGP AGE WEIGHTING PARAMETER α

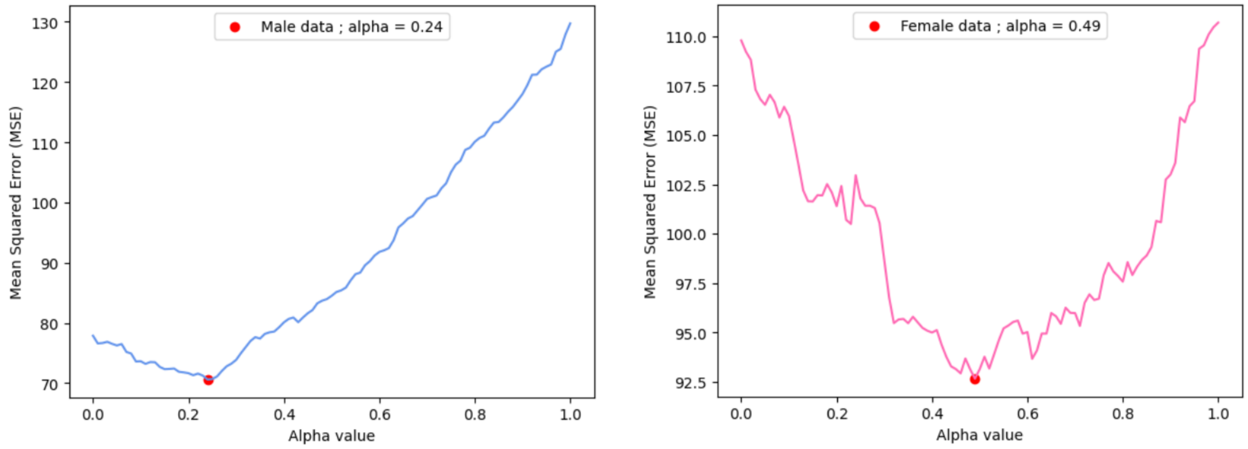

Figure 7. Sensitivity of prediction error to the SGP age weighting parameter α. Mean squared error (MSE) as a function of the fusion weight α for (left) male and (right) female cohorts. The weighting parameter α was selected by sweeping its value over the interval $[0, 1]$ and identifying the minimum of the error curve on the validation set. The curves illustrate the optimization landscape used to determine α. For males, the optimal value occurs near $\alpha = 0.24$, whereas for females it occurs near $\alpha = 0.49$.

5 HIGH PREDICTION ERROR CASES

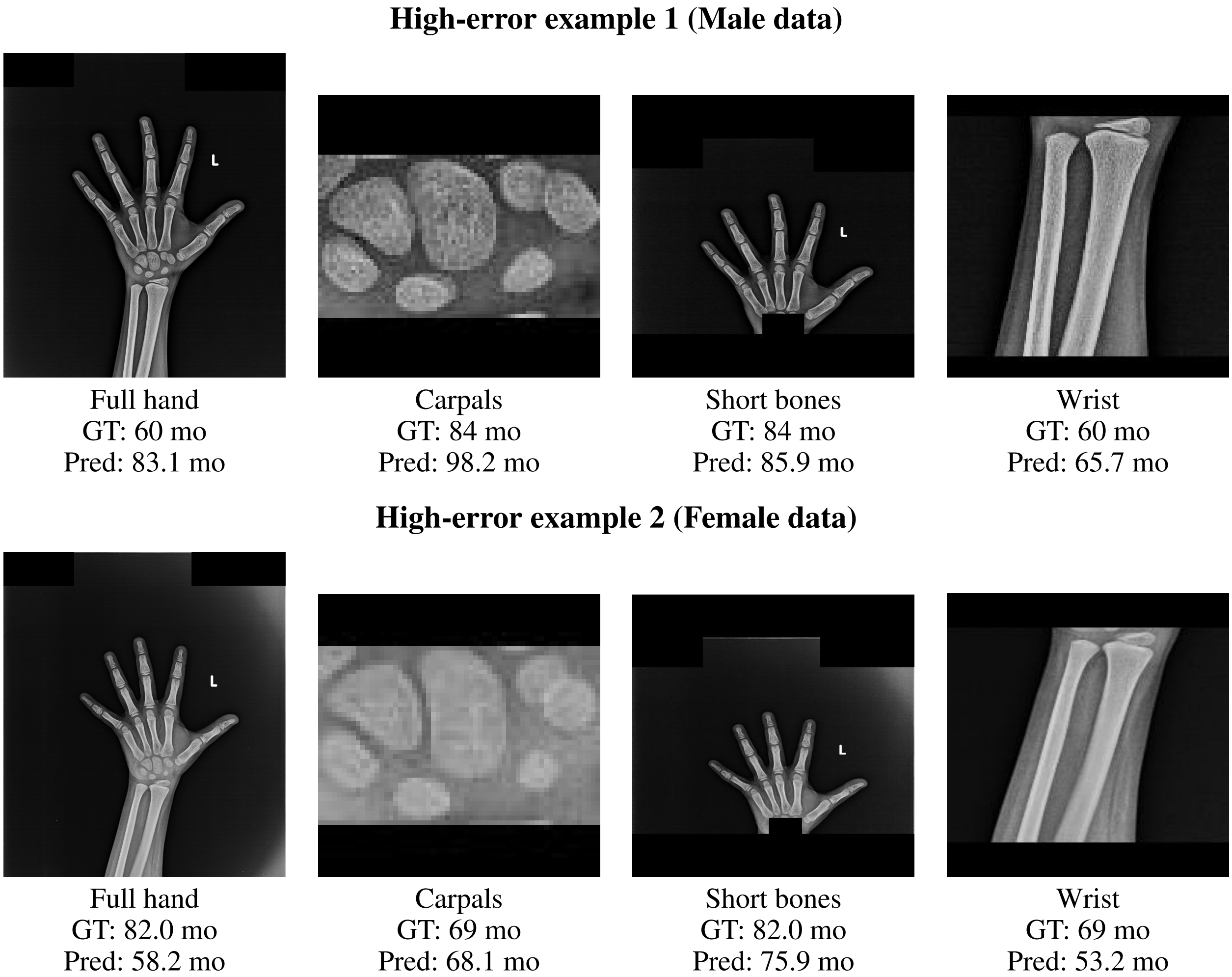

Figure 8. Representative high prediction error cases were identified based on the Bland–Altman analysis: specifically, radiographs for which the full-hand prediction error lay beyond two standard deviations from the mean difference were selected for detailed inspection. Notably, although the full-hand predictions exhibited large deviations from the reference bone age, several segmental predictions for the same radiographs showed substantially smaller errors.

REFERENCES

Chapke, R. (2023). *Segmentation of Pediatric Hand Radiograph Using UNet for Bone Aging*. Master's thesis, Indian Institute of Science Education and Research Pune. Available at: http://dr.iiserpune.ac.in:8080/xmlui/handle/123456789/7873
